# CACTUS: cancer image annotating, calibrating, testing, understanding and sharing in breast cancer histopathology

**DOI:** 10.1186/s13104-019-4866-z

**Published:** 2020-01-06

**Authors:** Alper Aksac, Tansel Ozyer, Douglas J. Demetrick, Reda Alhajj

**Affiliations:** 1 University of Calgary, Calgary, AB Canada; 2 TOBB University of Economics and Technology, Ankara, Turkey; 3 Laboratory Medicine, University of Calgary and Calgary Laboratory Services, Calgary, AB Canada; 4 Istanbul Medipol University, Istanbul, Turkey

**Keywords:** Medical image analysis, Breast cancer, Histopathology, Annotation, Grading

## Abstract

**Objective:**

Develop CACTUS (cancer image annotating, calibrating, testing, understanding and sharing) as a novel web application for image archiving, annotation, grading, distribution, networking and evaluation. This helps pathologists to avoid unintended mistakes leading to quality assurance, teaching and evaluation in anatomical pathology. Effectiveness of the tool has been demonstrated by assessing pathologists performance in the grading of breast carcinoma and by comparing inter/intra-observer assessment of grading criteria amongst pathologists reviewing digital breast cancer images. Reproducibility has been assessed by inter-observer (kappa statistics) and intra-observer (intraclass correlation coefficient) concordance rates.

**Results:**

CACTUS has been evaluated using a surgical pathology application—the assessment of breast cancer grade. We used CACTUS to present standardized images to four pathologists of differing experience. They were asked to evaluate all images to determine their assessment of Nottingham grade of a series of breast carcinoma cases. For each image, they were asked for their overall grade impression. CACTUS helps and guides pathologists to improve disease diagnosis with higher confidence and thereby reduces their workload and bias. CACTUS can be useful for both disseminating anatomical pathology images for teaching, as well as for evaluating agreement amongst pathologists or against a gold standard for evaluation or quality assurance.

## Introduction

Advanced image capturing and analysis in digital pathology has brought in more insight to pathologists and guided them in identifying and grading diseases. Staging and grading methods may differ for various types of cancer; the most commonly for breast cancer is Nottingham grading system [1, 2]. It evaluates three criteria, namely: nuclear pleomorphism, tubular formation, and number of mitotic figures in the most active areas; each is assigned a score from 1 to 3. The sum of these scores determines the grade of the analyzed breast cancer case.

Histopathological tissue analysis based on correct detection and annotation of nuclei, tubules and mitosis, is done manually by expert pathologists. This process requires considerable effort, expertise and experience. These skills are gradually gained over years by experiencing different cases in clinical duties and receiving feedback from both more experienced domain experts and the clinical courses of patients. Whereas visual interpretation leads to inter/intra-observer variability [3, 4], and some potential decreased reproducibility. These tools intend to increase the performance of pathologists regarding speed and accuracy [5]. Therefore, it is important to develop an automatic evaluation tool for quantitative and qualitative analysis to eliminating this disadvantage.

Despite the existence of several applications, histopathological examination of tissues is still a challenging problem. This is true because fixation, embedding, sectioning and staining steps in tissue preparation produce large amounts of artifacts and differences [6]. Also, the variability in size, shape, location, texture of a nuclei turn automated detection into a more complicated process.

To contribute to this highly essential and demanding domain, this paper describes a novel tool called CACTUS (cancer image annotating, calibrating, testing, understanding and sharing), which is to the best of our knowledge the first comprehensive tool intended to help and guide pathologists in their effort to improve disease diagnosis and thereby reduce their workload and bias.

CACTUS has an interactive machine learning approach to keep its model accurate and robust. To increase the confidence in the outcome of CACTUS and to turn it into a more attractive tool, it has been distinguished by integrating a social network construction and analysis component which guides a pathologist further based on the social network of his/her collaborators and co-authors. This leads to a recommendation system that highlights to a pathologist the discoveries by experts and influential collaborators and co-authors; this may lead to faster learning with higher confidence.

The social network between pathologists increases engagement and interaction by improving the comparability of the results obtained by people in different labs. This is another way to overcome subjectivity in decision-making. A breast cancer and its grade can be detected more accurately by combining machine learning and graph theory algorithms [7, 8] with image analysis.

## Main text

The main text of this paper is organized as follows. “Brief overview of existing similar tools” section is an overview of the existing similar tools which had been completed for medical image processing. “The user interface and functionalities” section presents the functionality and the user interface of CACTUS. “Conclusions” section highlights main remarks related to the conducted study.

### Brief overview of existing similar tools

Several tools and libraries have been developed for medical image analysis, both for commercial and research purposes. In this section, we briefly cover some of the popular tools described in the literature. A comparison of existing similar tools is given in Table 1.

**Table 1:** Comparison of existing similar tools

	CACTUS	ImageJ	CellProfiler	CellOrganizer	Labelbox	Dataturks
License	Open source, free	Open source, free	Open source, free	Open source, free	Commercially available	Commercially available
Type	Server	Client	Client	Client	Server	Server
Area selection	Manual labeling/automated detection	Polygonal	No	No	Polygonal	Polygonal
Multi-class	Yes	Yes	NA	NA	Yes	Yes
Version history	Yes	No	No	No	No	No
Interactive machine learning	Yes	NA	No	No	NA	NA
Analytics	Yes	Yes	Yes	Yes	NA	Yes
Quality assurance	Yes	No	No	No	No	No
Collaborative	Yes	No	No	No	NA	Yes

‘NA’ refers to either a system is not designed for the specific purpose, or well-known examples of this system for a specific purpose were not available when this article was written

The ilastik toolkit [9] is a simple tool for interactive image classification, segmentation and analysis. It is a free and open-source. Installers for Linux, macOS and Windows are available.

ImageJ [10] is an open-source image processing system designed for scientific multidimensional images. Installers for Linux, macOS and Windows are available. It is also a popular tool since it is highly extensible, with thousands of plugins and scripts for performing a wide variety of tasks, and a large user community.

CellProfiler [11] is free, open-source software designed to aid biologists without computer-vision training to quantitatively measure cell images automatically.

CellOrganizer [12] is a free and open-source tool. It builds generative models of the cellular organization by modeling the structure of a cell and a nucleus shape given high resolution 2D and 3D microscopy images.

Labelbox [13] and Dataturks [14] provide image annotation or segmentation tasks management, They are particularly useful when crowd-sourcing the annotations.

### The user interface and functionalities

**Fig. 1:**
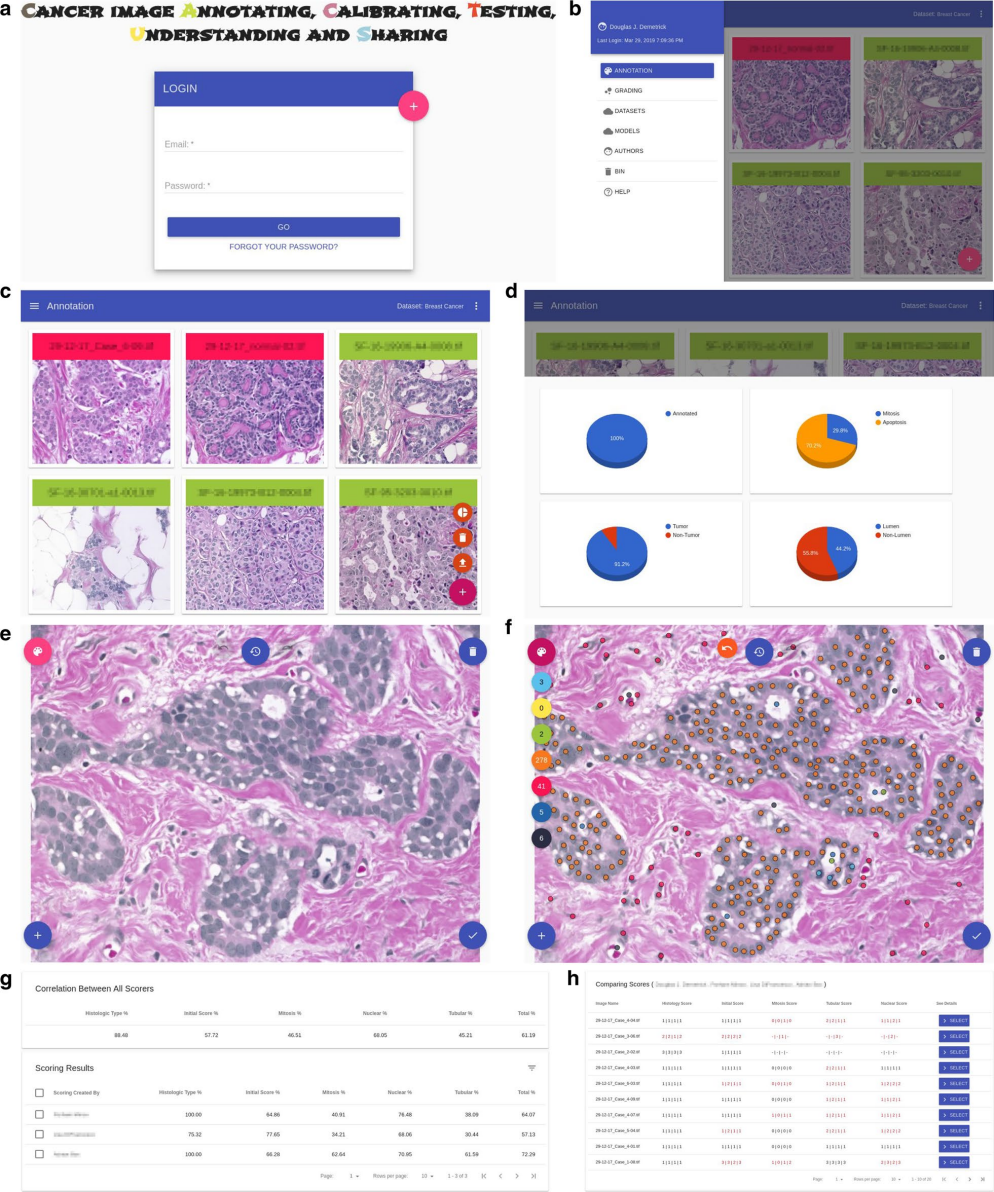
**a** Login screen, **b** menu screen, **c** annotation screen, **d** annotation details screen, editor screens for annotation are shown in **e** before annotation and **f** after annotation, grading screens for annotation are shown in **g** inter-observer agreement between users and **h** detailed comparison screen

CACTUS provides a simple user-friendly interface that helps users in their effort to improve disease diagnosis, and thereby reduces their workload and the bias among them. The CACTUS server has been implemented in Python using the Flask framework by running on top of MongoDB, which operates depending on Python environment and can run on multi-OS (including, Windows, macOS and Linux). The CACTUS website has been developed as a single-page application powered by JavaScript library AngularJS. It utilizes a simple, easy to use graphical interface (see the Login Screen in Fig. 1a).

The user can navigate in the web application by clicking the menu icon available on the top left corner. As a result, a navigation dialogue is displayed on the screen shown in Fig. 1b.

#### Annotation

When users log in, the list of images with a red flag (not annotated yet) is automatically loaded, see Fig. 1c. Alternatively, to reach this section, users can select the menu icon at the top left corner and choose annotation from the navigation menu (see Fig. 1b). After completing the assessment, the annotated images will be flagged green. Users may decide to stop and log off at any moment. Their progress and changes will be saved. They may come back later and resume from where they were left. There is a floating action button (FAB), a circular button at the bottom right corner, which triggers the primary action in the application’s UI such as upload, delete and details about annotations (see Fig. 1d).

In the annotation editor screen, users have several FABs positioned on the app’s UI. The annotation palette button is located at the top left corner. Users can choose their annotation types (such as mitosis, non-mitosis, apoptosis, tumor, non-tumor, lumen, non-lumen from top to bottom, respectively, as shown in Fig. 1e, f). Moreover, these annotations can be done automatically using specifically trained models for nuclei, mitosis and tubular predictions. The auto annotation button is located at the bottom left corner. The history button is positioned on the top at the center of the screen. Users can undo or redo their recent annotation actions. They can also load the previous version from other annotation sessions. Users can remove the annotations using the remove button located on the top right side of the screen. All changes can be saved by clicking the save button located at the bottom right side of the screen.

#### Grading

The grading screen has been designed very similar to the annotation screen. Users will see the selected list of images with a red flag (not scored yet). Once users complete their assessment, the images will be flagged green. Unless they are completed, the images will be blue. Also, users may decide to stop and log off at any moment. Their progress and changes will be saved, and their current case will be marked as unfinished with a blue flag. They can continue where they were left off.

We have evaluated the tool by presenting standardized images from the BreCaHAD dataset [5] to four surgical pathologists of varying practice interests in surgical pathology. They were first asked for their initial impression of the cancer grade from a single image. Then, they were asked about their impression of the individual grading criteria. The experience of the four participating pathologists is more than 15 years except one. Moreover, 3 out of 4 pathologists are surgical pathologists.

CACTUS permits the evaluation of individual answers as compared to a single *gold standard* and the agreement amongst the pathologists for each criterion. This information allows the identification of pathologists who are consistently evaluating standardized images or histological criteria differently compared to their cohort, thus allowing self-assessment, potential external intervention, and/or re-education of other participants in the quality assurance exercise.

Statistical analysis of the inter-observer agreement is shown in Fig. 1g, h. Figure 1g is the main screen for the statistical analysis where the top card shows the inter-observer agreement in Fleiss $\kappa$ values (multiple observers) between pathologists for given cases, and the bottom card includes a table to show the level of agreement between pairs of observers (one-to-one) based on Cohen $\kappa$.

Figure 1h is the detailed comparison screen in table format where each row represents the case and each cell includes scores from pathologists. The order of pathologists is given in the table title. If there is a difference between pathologists, the cell is highlighted with red color. The user can also see these differences in detail by clicking the *Select* button on the scoring screen.

**Fig. 2:**
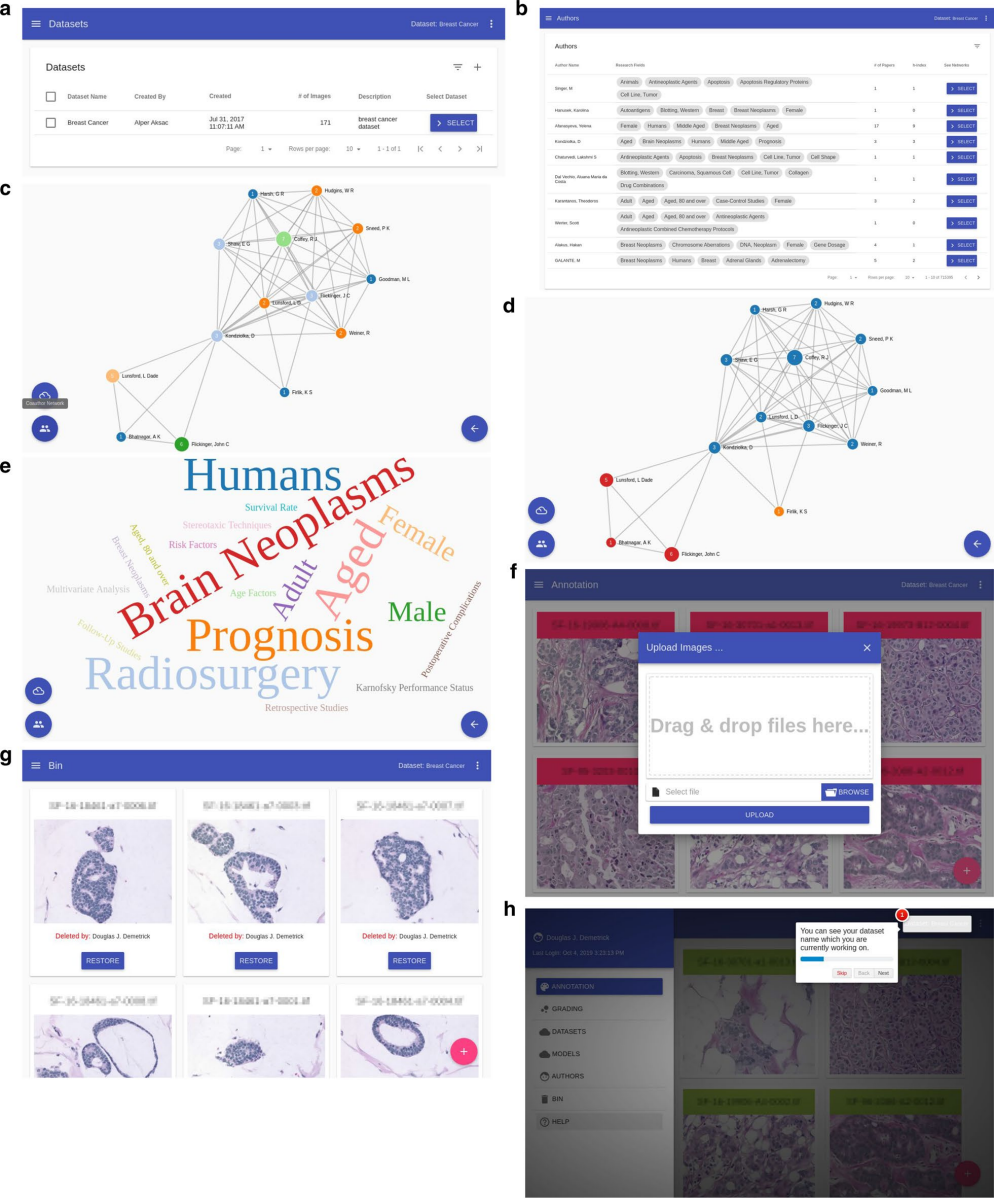
**a** Datasets screen, **b** authors screen, network and word cloud screens for authors are shown in **c** co-author network, **d** communities in co-author network and **e** word cloud in research fields, other screens are shown in **f** upload, **g** bin/recover and **h** help

#### Datasets and models

As shown in Fig. 2a, users can choose from the table any available dataset to annotate and/or grade by clicking the *Select* button. Users can create a new database by clicking the *plus sign* (“+”) icon on the top right corner of the table. It is also possible to search/delete by clicking the *filter* icon next to the plus sign icon.

The models screen has been designed very similar to the datasets screen. In this screen, users can select models to be used in the annotation editor screen where if the quality of predictions is very poor, the model can be re-trained more efficiently by incorporating human feedback to provide more accurate results. If provided, the feedback does not improve the overall outcome, users still can choose older models from this table. They can even delete unsuccessful models from the system.

#### Authors

As shown in Fig. 2b, the “Authors” function allows users to search for authors or colleagues who are conducting breast cancer research. Users can see their co-authors’ network or their active research fields by clicking the *Select* button. The co-authorship network represents collaborations between authors who co-published papers. It is also possible to apply community detection techniques to examine the structure of the co-authorship network. The author’s active research fields are shown in the word cloud where font size reflects the importance of each tag (Fig. 2c–e).

#### Others

The upload screen allows users to upload case images to the already selected database by either drag and drop or by selecting them from a folder. The Bin/Recover screen allows users to recover images that were removed and not included in the annotation process. The help function shows and explains to users how to use the application step by step, see Fig. 2f–h.

#### Conclusions

We have designed and tested a tool that could be used for education, evaluation and quality assurance purposes in a wide spectrum of surgical pathology practices. The proposed tool will help and guide pathologists in their effort to improve disease diagnosis and thereby reduce their workload and the bias among them. A breast cancer and its grade can be detected more accurately by combining machine learning and graph theory algorithms with the help of image analysis.

Future work will focus on improving the annotation module with advanced features such as highlighting nuclei boundaries or other structures while selecting them in the annotation process. Moreover, adding and developing 3D histology-based methods to the proposed system will allow presenting improved visualizations and results in the histopathology image analysis.

## Limitations

Although the development of the application is complete from the user’s perspective, the performance of the code can still be improved in future versions. CACTUS has initially been designed for users with minimal training. Later on, it will be developed in a more advanced user-friendly manner. Besides, CACTUS will be significantly improved by adding new features and further customization of the visualizations. The application currently supports only breast cancer analysis. The planned task is to extend the application usability to other diseases and types of cancer, starting with prostate cancer.

## Data Availability

CACTUS is released under the MIT license and is available at GitHub (https://github.com/alperaksac/cactus).

## Abbreviations

CACTUS: cancer image annotating, calibrating, testing, understanding and sharing
BreCaHAD: breast cancer histopathological annotation and diagnosis
FAB: floating action button
